# Recommended level of physical activity and health-related quality of life among Japanese adults

**DOI:** 10.1186/1477-7525-5-64

**Published:** 2007-11-28

**Authors:** Ai Shibata, Koichiro Oka, Yoshio Nakamura, Isao Muraoka

**Affiliations:** 1 Research Institute for Elderly Health, Comprehensive Research Organization, Waseda University, Tokyo, Japan; 2 Faculty of Sport Sciences, Waseda University, Saitama, Japan

## Abstract

**Background:**

The benefits of a recommended level of physical activity on physiological health indicators such as morbidity and mortality are well-accepted, but less research has addressed whether or not the association between the recommended level of physical activity and a health-related quality of life (HRQOL) exists in the Japanese population. Thus, the present study examined whether the recommended physical activity would be associated with HRQOL in the general Japanese middle-aged population.

**Methods:**

Data were obtained from 1211 male and female respondents (39.4 ± 10.9 year, mean ± SD) from an Internet-based survey of registrants of an Internet research service. Physical activity level was estimated from the short form of the International Physical Activity Questionnaire. HRQOL was assessed with the Medical Outcomes Survey Short Form-8 questionnaire (SF-8). Based on the current national guidelines for exercise in Japan, respondents were divided into a recommended group, an insufficient group, and an inactive group according to their estimated weekly physical activity level. Multivariate analyses of covariance were utilized.

**Results:**

Across both genders, the recommended group had significantly higher physical functioning (PF) scores than the inactive group (p < .05). Additionally, across both genders, the recommended group had significantly higher general health perception scores than the insufficient and inactive groups (p < .05). The recommended group had significantly higher vitality scores than the insufficient and inactive groups in males, and higher than only the inactive group in females (p < .05). The insufficient group had significantly higher PF scores than the inactive group across both genders (p < .05). The recommended group had significantly higher physical component scores than the inactive group (p = .001).

**Conclusion:**

Individuals who attained the recommended level of physical activity had better scores on some dimensions of HRQOL than those who did not, suggesting that the recommended level of physical activity may be applicable not only to the physiological objective outcomes but also to some dimensions in both the physical and mental aspects of HRQOL.

## Background

The Healthy Japan 21 campaign aims to prevent chronic diseases, increase the quality of life (QOL), and expand years of healthy life for all persons in Japan [1]. The promotion of physical activity is now recognized as an important component of such a national disease prevention policy. The benefits of physical activity on health are well established. Regular physical activity is associated with a decreased incidence of cardiovascular disease, stroke, and diabetes mellitus; reduced coronary artery disease risk factors such as hypertension, dyslipidemia, and obesity; and improved mood states, including depression and anxiety [2,3].

Health-related QOL (HRQOL) refers to the perception of overall satisfaction with life and involves the measurement of functional status in the domains of physical, cognitive, emotional, and social health, and becomes a fundamental assessment in understanding the health status of a population [4,5]. As of now, the beneficial effect of exercise intervention on HRQOL was mainly found in special populations [6-9]. According to the US Surgeon General's Report [2], regular physical activity appears to improve HRQOL by enhancing psychological well-being and by improving physical functioning.

In Japan, the guidelines and recommendations for physical activity and exercise were published in 2006 as part of health promotion [10]. In the current guidelines, an increase in daily physical activity above three metabolic equivalents (METS) receives greater emphasis as compared with the former traditional exercise guidelines, which recommended at least 20 to 30 minutes of moderate-intensity exercise and walking on most days of the week [11]. The present recommendation states that every adult should accumulate 23 METS/hour/week in order to prevent chronic diseases and obtain numerous health benefits [10].

The benefits of the recommended level of physical activity on physiological health indicators such as morbidity and mortality are wellaccepted [2,3]. In addition, previous studies found that exercise intervention improved HRQOL in those with chronic diseases [6-9]. However, the association between the recommended level of physical activity and HRQOL in the general Japanese population was still not obvious. A few previous researchers in other countries found that the recommended level of physical activity might affect HRQOL by influencing two main components: physical functioning and wellbeing [2,12,13]. Nevertheless, few studies have examined the association between the current Japanese recommendation for physical activity and HRQOL in the general Japanese population. Thus, the present study was proposed to examine whether the recommended level of physical activity would be associated with HRQOL in the general Japanese middle-aged population.

## Methods

### Participants

The current study used a sample comprising 1211 male and female respondents to a cross-sectional survey on the association between sports and health. The survey was provided by the Japan Sports Industries Federation in September 2006. The set sample size and parameters were approximately 1200 male and female adults aged between 20 and 59 years, with an equivalent number of males and females in each age bracket. Of approximately 230,000 registrants for the Internet research service, potential respondents were randomly selected in accordance with the set sample size and parameters and were invited to participate in an Internet-based survey via e-mail. Internet-based questionnaires were placed in a protected area of a web site and the potential respondents received the URL in an invitation e-mail. Reward points for the Internet service were provided as incentives for participation. All respondents voluntarily completed and signed an online Institutional Review Board-approved letter of informed consent and demographic data information. The demographic data included gender, age, marital status, educational level, and household income level. In addition, the following measures were administered.

### Measurements

#### Physical Activity

The short version of the International Physical Activity Questionnaire (IPAQ) was utilized to estimate the amount of physical activity that the participants engaged in. The IPAQ has been used in several countries [14]. This self-administered questionnaire was designed to be utilized by adults aged between 18 and 65 years. It identifies the frequency and duration of walking, moderate and vigorous physical activity, and sedentary activity during the past week [14]. The one-week test-retest reliability of the short, self-administered Japanese version of IPAQ is good (Spearman r = 0.72–0.93). The criterion validity for the Japanese version of IPAQ against the accelerometer is acceptable (Spearman r = 0.39) [15]. However, the validity of the Internet-based Japanese version of IPAQ has not yet been tested.

The short-form data were utilized to estimate the total weekly physical activity level (METS/hour/week) by weighting the reported hours per week within each of the three activity categories: low, moderate, and high by MET energy expenditure estimates assigned to each category of activity. The current national guidelines for exercise in Japan recommend 23 METS/hour/week of physical activity [10]. Based on the estimated total weekly physical activity level, respondents were assigned to one of three (mutually exclusive and exhaustive) groups. Individuals who reported no physical activity were assigned to the inactive group; those who reported physical activity that was less than the recommended level but greater than nothing were assigned to the insufficient group; and those who reported 23 or more METS/hour/week of physical activity were assigned to the recommended group.

#### HRQOL

The Japanese version of the Medical Outcomes Study (MOS) Short Form 8-Item Health Survey (SF-8) was administered to assess the HRQOL. The SF-8 consists of 8 items and is the most recent version of the MOS short form health surveys. Similarly to the MOS 36-item short form health survey (SF-36), the SF-8 is divided into an 8-dimension health profile: physical functioning (PF), role functioning- physical (RP), bodily pain (BP), general health perception (GH), vitality (VT), social functioning (SF), role functioning-emotional (RE) and mental health (MH), and comparable estimates of summary scores for the physical and mental components of health (PCS, MCS). Each item of the SF-8 is assessed by a 5- or 6-point Likert scale. The 8-domain scaled scores range from 0 to 100, with 100 representing optimal health and functioning [16]. The 8-domain summary scores, PCS and MCS, have been normalized to the Japanese population. The reliability of the Japanese version of the SF-8 by an alternate-forms method was adequate (Spearman r = 0.70–0.88) [16,17]. The Japanese version of the SF-8 meets the standard criteria for content and the construct and criterion validity [17]. The practical advantage of SF-8 is briefly to assess and directly compare the eight scores produced by the SF-36. The correlation coefficient of each 8-domain scale score between SF-8 and SF-36 was strong (Spearman r = 0.56–0.87) [17]. The validity of the original Internet-based English version of the SF-8 was examined by comparing the results obtained via the Internet, through a telephone interview, and a mail survey. All eight dimensions and two summary scores obtained via the Internet were significantly lower than those obtained by telephone interview and comparable to those obtained by the mail survey, with the exceptions of RP, GH, RE, and PCS [17]. Nevertheless, the validity of the Internet-based Japanese version of the SF-8 has not yet been investigated.

### Statistical analysis

For the analysis, respondents with incomplete information for all study variables (n = 39) and extreme estimated physical activity level from IPAQ (n = 20) were excluded. Consequently, 1152 individuals were available for data analysis. A chi-squared test was utilized to compare differences in categorical variables among the physical activity groups. Additionally, a multivariate analysis of variances (MANOVA) was conducted to determine the differences in the SF-8 measures among each demographic group. The univariate analyses and Tukey's post hoc tests were performed following significant multivariate effects.

The primary analysis was stratified by gender. Multivariate analyses of covariance (MANCOVAs) were utilized to examine differences in multidimensional scales of the SF-8, with physical activity levels as the between-group factor and age, marital status, household income level, and educational level as covariates. Significant multivariate effects were followed up with the Bonferroni-adjusted univariate ANOVA. The alpha level was set at .05. The Statistical Package for Social Science (SPSS) for Windows 14.0 was utilized to compute the statistics [18].

## Results

### Basic characteristics of respondents

**Figure 1:**
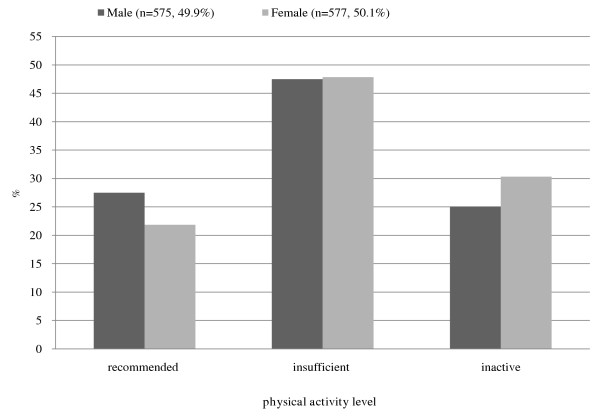
Prevalence of physical activity level by gender.

In the present study, 575 males and 577 females were classified into three groups according to physical activity level (Figure 1). The average age was comparable across the three physical activity groups. Males (n = 158, 27.47%) were more likely to meet the recommended level of physical activity than females (n = 126, 21.84%). Similarly, females (n = 175, 33.03%) were more likely to be inactive than males (n = 144, 25.04%). Those differences seemed to be driven by the 20- and 50-year age groups. Although the number of those who attain the recommended level and who are deemed inactive was relatively similar in the 30- and 40-year age groups across gender, the likelihood of engagement in the recommended level of physical activity in females in the 20- and 50-year age groups (22.07%, 25.69%) was significantly lower than those in males in corresponding groups (36.99%, 30.50%). Additionally, 35.2% of younger (20–29 years) and 30.6% of older (50–59 years) respondents in females were physically inactive, whereas the same results were observed in 22.6% and 22.0% in males, respectively. The number of those who engaged in an insufficient level of physical activity was similar across genders (male: n = 274, 47.48%; female: n = 276, 47.83%). The respondents who met the recommended level of activity were less likely to be in the 30-year age group for both males and females. Table 1 presents the demographic characteristics of the study population stratified by physical activity level and gender.

**Table 1:** Respondent characteristics among three physical activity groups stratified by gender

	Male (n = 575, 49.9%)			Female (n = 577, 50.1%)		
	
	recommended	insufficient	inactive	recommended	insufficient	inactive
N	158	273	144	126	276	175
%	27.48	47.48	25.04	21.84	47.83	30.33
Mean Age (SD)	38.72(12.16)	40.08(10.75)	39.22(9.90)	40.12(11.86)	39.46(10.49)	38.34(10.94)
Age group N (%)						
20–29	54 (36.99)	59 (40.41)	33 (22.60)	32 (22.07)	62 (42.76)	51 (35.17)
30–39	28 (19.45)	77 (53.47)	39 (27.08)	25 (17.24)	75 (51.72)	45 (31.03)
40–49	33 (22.92)	70 (48.61)	41 (28.47)	32 (22.38)	76 (53.15)	35 (24.47)
50–59	43 (30.50)	67 (47.52)	41 (21.98)	37 (25.69)	63 (43.75)	44 (30.56)
Maital status N (%)						
married	90 (26.63)	160 (47.34)	88 (26.04)	89 (22.70)	181 (46.17)	122 (31.12)
unmarried	68 (28.69)	113 (47.68)	56 (23.63)	37 (20.00)	95 (51.35)	53 (28.65)
Educational level N (%)						
4-years university or greater	103 (28.30)	178 (48.90)	83 (22.80)	43 (22.51)	104 (54.45)	44 (23.04)
2-years university or equivalent	11 (15.07)	36 (49.32)	26 (35.62)	45 (20.55)	109 (49.77)	65 (29.68)
high school or junior high school	43 (32.09)	57 (42.54)	38 (25.37)	38 (23.90)	58 (36.48)	63 (39.62)
other	1 (25.00)	2 (50.00)	1 (25.00)	0 (00.00)	5 (62.50)	3 (37.50)
Household income level N (%)						
<3,000,000 yen	27 (31.40)	39 (45.35)	20 (23.26)	18 (20.22)	40 (44.94)	31 (34.83)
<5,000,000 yen	40 (26.49)	64 (42.38)	47 (31.13)	41 (23.03)	71 (39.89)	66 (37.08)
<7,000,000 yen	28 (24.78)	54 (47.79)	31 (27.43)	29 (24.17)	63 (52.50)	28 (23.33)
<10,000,000 yen	38 (25.50)	78 (52.35)	33 (22.15)	22 (18.18)	65 (53.72)	34 (28.10)
<15,000,000 yen	19 (32.76)	31 (53.45)	8 (13.79)	13 (23.64)	30 (54.55)	12 (21.82)
≥15,000,000 yen	6 (33.33)	7 (38.89)	5 (27.78)	3 (21.43)	7 (50.00)	4 (28.57)

### Effects of demographic characteristics on HRQOL

Regarding the 8-domain scales scores, a one-way MANOVA was conducted to examine the group differences in the SF-8 measures for each demographic variable. The multivariate effects for gender (Wilk's λ = .954, p = .000), marital status (Male: Wilk's λ = .958, p = .002; Female: Wilk's λ = .964, p = .007), and age level (Male: Wilk's λ = .921, p = .004; Female: Wilk's λ = .929, p = .012) were significant.

With respect to gender, the univariate analyses indicated significant differences in RP, BP, GH, SF, and MH. Males had significantly higher RP, BP, GH, SF, and MH than females. For marital status, the univariate analyses indicated significant differences for RE and MH in males and MH in females. The married males had significantly higher RE and MH scores than the unmarried males. Married females also had significantly higher MH than the unmarried females. With regard to age level, the univariate analyses indicated significant differences for BP, SF, RE, and MH in males and MH in females. The RE for the 50-year age group was significantly higher than for the other three groups (20-, 30-, and 40-year age groups); the MH for the 50-year age group was significantly higher than that for the 20- and 30-year age groups. The MH for the 20-year age group was significantly lower than that for the 40- and 50-year age groups.

Likewise, gender (Wilk's λ = .989, p = .002), age level (Male: Wilk's λ = .946, p = .000; Female: Wilk's λ = .962, p = .001), and marital status (Male: Wilk's λ = .964, p = .000; Female: Wilk's λ = .977, p = .001) achieved statistical significance in the multivariate effects of PCS and MCS. The males had significantly higher PCS scores than the females. The married males had significantly higher PCS and MCS scores than the unmarried males. The married females also had significantly higher MCS than the unmarried females. In the case of the males, the MCS for the 50-year age group were significantly higher than those for the 20- and 30-year age groups; and the PCS for the 20-year age group were significantly higher than those for the 40- and 50-year age groups. In females, the MCS for the 20-year age group were significantly lower than those for the 40- and 50-year age groups.

### Effects of physical activity level on HRQOL

Regarding the 8-domain scales scores, the between-physical activity group differences were investigated among all demographic variables. For both genders, all eight domains of the SF-8 were slightly higher in the recommended group than in the inactive group, with the exception of BP in females. However, the difference in scores between the recommended and inactive groups was relatively small, ranging from 3.11 to 0.59 points for males and 3.06 to 0.49 points for females. Moreover, for both genders, the differences between the recommended and insufficient groups were much smaller than those between the recommended and the inactive groups.

The physical activity groups were found to differ significantly only in regard to age, [Male: F(8.561) = 3.788, p = .000; Female: F(8.563) = 2.592, p = .009]. Marital status, household income level, and educational level failed to achieve statistical significance in the multivariate model. Therefore, only age was included as a covariate in all subsequent analysis. A one-way MANCOVA was conducted to examine the group differences in the SF-8 measures. The multivariate effects for physical activity level were significant (Male: Wilk's λ = .943, p = .007; Female: Wilk's λ = .923, p = .000). The univariate analyses indicated significant differences for PF [Male: F(2.568) = 6.62, p = .001; Female: F(2.570) = 7.59, p = .001], GH [Male: F(2. 568) = 7.09, p = .001; Female: F(2. 570) = 5.55, p = .004], and VT [Male: F(2. 568) = 8.36, p = .000; Female: F(2. 570) = 5.66, p = .004] in both genders. Across both genders, the recommended group had significantly higher PF scores than the inactive group. Additionally, across both genders, the recommended group had significantly higher GH scores than the insufficient and inactive groups (p < .05). Moreover, the males in the recommended group had a significantly higher VT score than those in the insufficient and inactive groups of males, which was only higher than those for females in the inactive group (p < .05). Across both genders, the insufficient group had significantly higher PF than the inactive group (p < .05).

With regard to PCS and MCS, only age level achieved statistical significance in the multivariate model [Male: F(2.567) = 8.724, p = .000; Female: F(2.569) = 7.619, p = .001]. Thus, only age was included as a covariate in all subsequent analyses. A one-way MANCOVA was utilized to examine the group differences in PCS and MCS. The multivariate effects for physical activity level were significant only in males (Wilk's λ = .975, p = .005). The univariate analyses indicated significant differences for PCS [F(2.568) = 6.600, p = .005]. The recommended group had significantly higher PCS scores than the inactive group (p = .001). All significant differences persisted, despite the adjustment of age. The results of the MANCOVAs and univariate analyses for physical activity level and HRQOL measures were presented in Table 2.

**Table 2:** Unadjusted HRQOL measures in respondents among physical activity groups stratified by gender

	physical activity group				
			
Male mean (SD)	recommended	insufficient	inactive	*F*^§^	^#^
PF	50.82 (4.31)	49.74 (6.89)	47.96 (8.99)	6.61**	b**, c*
RP	50.87 (4.57)	50.24 (5.49)	49.42 (6.25)	2.64	
BP	51.64 (7.57)	51.69 (7.77)	50.37 (8.45)	1.48	
GH	50.36 (6.96)	47.99 (6.68)	47.89 (6.86)	7.02**	a**, b**
VT	50.84 (6.74)	48.57 (6.85)	47.73 (7.51)	8.34***	a**, b**
SF	48.83 (8.49)	48.79 (7.93)	47.24 (8.81)	1.91	
RE	48.83 (7.46)	48.83 (6.47)	48.27 (6.75)	0.41	
MH	49.16 (7.68)	48.72 (7.00)	48.46 (7.03)	0.56	
PCS	50.65 (4.89)	49.38 (6.60)	48.00 (6.68)	6.59**	b**
MCS	47.56 (8.58)	47.30 (7.31)	47.04 (7.22)	0.32	

Female mean (SD)					

PF	50.44 (4.57)	49.40 (6.24)	47.38 (9.36)	724**	b**, c*
RP	49.45 (5.37)	49.27 (6.16)	48.83 (7.26)	0.29	
BP	48.42 (8.06)	48.89 (8.18)	48.91 (8.55)	0.21	
GH	49.47 (7.30)	47.40 (7.15)	46.64 (7.10)	5.51*	a*, b**
VT	50.39 (6.40)	48.84 (6.90)	47.60 (7.29)	5.59*	b**
SF	47.23 (8.19)	47.21 (8.13)	46.58 (8.98)	0.14	
RE	48.89 (6.87)	48.08 (7.46)	48.01 (7.05)	0.56	
MH	47.76 (7.22)	46.85 (7.88)	47.16 (7.40)	0.72	
PCS	49.05 (6.42)	48.52 (6.67)	47.17 (7.84)	2.95	
MCS	47.13 (7.74)	46.20 (7.99)	46.46 (7.43)	0.63	

HRQOL: Health related quality of life scale, Short Form-8

PF: Physical functioning, RP: Role physical, BP: Bodily pain, GH: General health, VT: Vitality, SF: Social functioning, RE: Role emotional, MH: Mental health, PCS: Physical component summary, MCS: Mental component summary

^§ ^comparison in multidimensional scales of SF-8 among physical activity levels with covariate of age, marital status, educational level and income level

^# ^Bonferroni-adjusted univariate multiple comparison

a: recommended vs. insufficient, b: recommended vs. inactive, c: insufficient vs. inactive

*** p < .000 ** p < .001 *p < .05

## Discussion

The current investigation was designed to examine whether or not the recommended level of physical activity would be associated with HRQOL in the general middle-aged Japanese population. Meeting the recommended level of physical activity was associated with better scores on GH, VT, and PCS in males, and only on GH and VT in females, even after the adjustment of age and socioeconomic status. Additionally, engaging in physical activity, even at insufficient levels, had a positive effect on the perception of PF in both genders. The researchers of the current study suggest that engaging in the recommended level of physical activity appears to be positively related to some dimensions in both the physical and mental aspects of HRQOL.

The current study is, perhaps, the first to examine the association between the recommended levels of physical activity and HRQOL in Japan. Previously, foreign researchers also found that the recommended levels of physical activity were positively associated with one or more dimensions of HRQOL. Vuillemin et al. [13] found that those who attained the recommended physical activity level scored significantly higher in almost all dimensions of SF-36 than those who did not attain the recommended level. In particular, the PF, GH, VT, SF, and MH were critically affected by the recommended level of physical activity. Brown et al. [12] also investigated the cross-sectional effects of recommended levels of physical activity on HRQOL. In this study, HRQOL was evaluated by asking questions about the number of physically and mentally unhealthy days experienced. The number of adults who met the recommended level of physical activity and reported 14 or more unhealthy days during the past 30 days was found to be sufficiently lower than the number of those who did not meet the recommended level of physical activity.

In prior cross-sectional studies similar to the current study, Laforge et al. [19] investigated using the association between the stage of readiness to exercise and HRQOL assessed with the SF-36. The stage was found to be significantly related to all dimensions of HRQOL; notably, a stronger association was observed in PF, GH, and VT dimensions. Wendel-Vos et al. [20] and Morimoto et al. [21] examined the relationship between the amount of physical activity and HRQOL. Wendel-Vos et al. [20] found a positive association between PF, GH, and VT of the SF-36 and time spent for leisurely physical activity (h/week). Morimoto et al. [21] also found that a greater amount of physical activity (kcal/week) was positively correlated with higher scores for all domains of the SF-36.

In the current study, the physical aspects of HRQOL, such as PF and GH, seemed to be more closely associated with the amount of physical activity than with mental aspects. This finding is consistent with several previous studies [19,20]. Although the perception of vitality-measuring the degree of energy, pep, or tiredness experienced-is classified as a mental health component in the SF-8 and the SF-36, it has a complex construction and is moderately correlated with both mental and physical health functioning [4]. Brown et al. [12] found that the number of physically unhealthy days was more strongly correlated with physical activity as compared with that of mentally unhealthy days in the general US population. The objective benefits on physical activity, such as a decreased risk of morbidity, may be directly reflected in the perception of physical health among respondents.

The findings of the current study differed from the previous studies with regard to the mental aspect of HRQOL [13,19-22]. The present study did not observe the association between all dimensions in the mental aspects of SF-8 and the recommended level of physical activity, with the exception of VT. Although the results reported in previous literature on the association between physical activity and mental aspects on the HRQOL are still somewhat controversial, numerous studies have been conducted on the effects of physical activity and exercise on the reduction of the symptoms of depression and anxiety [2,23]. Vuilleimn et al. [13] reported on the association between the perception of psychological well-being, such as VT, SF, and MH on the SF-36, and the recommended level of physical activity. Moreover, Morimoto et al. [21] have found that the mental aspects of HRQOL increased in proportion to the amount of physical activity, suggesting that the level of the current Japanese recommendation of physical activity on health promotion may be lower than the threshold of physical activity required to demonstrate a measureable impact on the mental aspects of HRQOL. Additionally, Laforge et al. [17] found that the longer the period for those who engaged in exercise at or above the recommended level, the more positive are the associations with higher mental dimensions of SF-36 in a period-dependent manner. This indicates that not only the amount but also the period of physical activity engaged in, which was not examined in the current study, may be one of the key factors influencing the mental aspect of HRQOL.

The current investigation had a number of limitations. First, the analysis was cross-sectional, making the determinations of cause and effect impossible to identify. Next, the physical activity level was administered using only the self-reported questionnaire; therefore, an inaccurate estimation of the physical activity level and recall bias are unavoidable. Moreover, the current study was conducted via the Internet. Eysenbach et al. [24] indicated that the issues of generalizability, mainly due to selection bias, were important considerations due to the nonrepresentative nature of the Internet population and the self-selection of participants being surveyed. Rhodes et al. [25] mentioned that younger, more educated, and higher income individuals have greater access to the Internet. Additionally, people are more likely to respond to a survey if they have an interest in the content of the questions or are attracted by the incentives offered for participation [24-26]. Therefore, the basic characteristics of respondents may possibly be biased, implying that the findings under such a setting may not be sufficiently applicable to the general Japanese middle-aged population. Also, the Internet-based Japanese version of IPAQ and the SF-8 were not previously validated for the Internet use. Thus, the results of the physical activity level and HRQOL administered via the Internet may be less accurate than those obtained by other validated methods such as the telephone interview and the self-administered survey. Additionally, chronic diseases or chronic conditions were not included as covariates in the current study, which may have been one of the factors leading to differential significant domains in the current study from those in prior studies. Those conditions are considered to be negatively correlated with HRQOL [27,28]. For example, Alonso et al. [27] reported that arthritis had a significant negative impact on both the PCS and MCS of the SF-36 in Japan. Therefore, those covariates should have been controlled.

## Conclusion

In summary, individuals who attained the recommended level of physical activity had better results on some dimensions of HRQOL than those who did not, suggesting that the current Japanese recommendation for physical activity may be applicable not only to physiological objective outcomes but also to HRQOL. If the perception of physical functioning and psychological well-being are improved through an increase in the physical activity level, it is sufficiently important to plan public health interventions designed to prevent a sedentary lifestyle and to promote physical activity. The current study highlights the need for future researchers to determine more accurately the association between HRQOL and the recommended physical activity level using a larger sample size. In addition, HRQOL may possibly be related to other characteristics and correlates of the Japanese population. Thus, a clarification of the characteristics and correlates possessed by individuals who meet the recommended level of physical activity, related to HRQOL, is needed to specify the target population in order to provide interventions for promoting physical activity. Moreover, to improve HRQOL, more effective interventions for physical activity promotion, which match the needs and expectations of the target population, should be developed in order to increase engagement in regular physical activity and exercise.

## List of abbreviations

QOL: quality of life; HRQOL: health-related quality of life; METS: metabolic equivalents; IPAQ: International Physical Activity Questionnaire; MOS: Medical Outcomes Study; SF-8: 8-Item Short-Form Health Survey; SF-36: 36-item short form health survey; PF: physical functioning; RP: role functioning physical; BP: bodily pain; GH: general health perception; VT: vitality; SF: social functioning; RE: role functioning emotional; MH: mental health; PCS: physical components of health

MCS: mental components of health; ANOVA: analysis of variance; MANCOVAs: Multivariate analyses of covariance; SPSS: Statistical Package for Social Science

## Competing interests

The author(s) declare that they have no competing interests.

## Authors' contributions

AS participated in the design of the study, performed the statistical analysis, and drafted the manuscript. YN and IM participated in the sequence alignment and helped to draft the manuscript. OK conceived of the study, and participated in its design and coordination and helped to draft the manuscript. All authors read and approved the final manuscript.
